# Oral Presentations

**DOI:** 10.1080/13577140120097058

**Published:** 2001-11


### ORAL PRESENTATIONS

### 040 Her-2/Neu Staining in Osteosarcoma: Association with Increased Risk of Metastasis

Holly Zhou, Rob Goldsby, R. Lor Randall, Lynn Smith, Cheryl M. Coffin
*Departments of Pathology, Pediatrics, Orthopedics, and Radiation Oncology, University of Utah*

*Objective:* Her-2/Neu expression in high grade osteosarcomas (OSC) has been proposed as an indicator of poor prognosis. This study correlates Her-2/ Neu staining in OSC with tumor progression and metastasis.
*Methods:* Her-2/Neu expression in 22 pre-treatment OSC and 12 pulmonary metastatic OSC was assessed by standard immunohistochemical techniques on formalin-fixed paraffin embedded tissue. The Her-2/Neu staining pattern, outcome and pulmonary metastases were analyzed.
*Results:* 10/22 pretreatment OSC displayed cytoplasmic Her-2/ Neu reactivity in more than 25% of tumor cells. Among these, 70% (7/10) patients had pulmonary metastases (4 at presentation, 3 later). In addition, focal cytoplasmic Her-2/Neu reactivity with more intense staining in foci with chondroblastic differentiation was identified in 7/22 pre-treatment OSC. 5/7 patients in this group had pulmonary metastases in a minimal follow-up of 4 years. The pattern of Her-2/Neu staining in pulmonary metastatic OSC is identical to that in primary OSC.
*Conclusion:* Cytoplasmic staining of Her-2/Neu in pre-treatment OSC correlates with increased risk of pulmonary metastasis. The Her-2/Neu positive cells may represent a chemoresistant, biologically aggressive subpopulation in OSC.

### 087 Reconstruction of the Pelvis Following Resection of Tumors about the Acetabulum

R. L. Satcher, Jr.[1], R. J. O'Donnell[1], J. O. Johnston[1]
*[1]University of California, San Francisco Department of Orthopaedic Surgery UCSF Comprehensive Cancer Center Orthopaedic Oncology Service 1701 Divisadero Street Suite 280, [2]The Permanente Medical Group, Inc. Department of Musculoskeletal Oncology 1200 El Camino Real*

*Objective:* Periacetabular resections for primary malignancies and metastatic disease require reconstruction to restore weight-bearing along anatomic axes. Without reconstruction, patients are unable to ambulate independently, and are left with a disfigured pelvis and a shortened limb. This abstract describes a reconstruction technique using rebar, cement, and autoclaved autografting, in combination with total hip arthroplasty, after resection of primary sarcomas of the pelvis.
*Methods:* This study retrospectively reviewed the results of 15 patients at 2 institutions who had surgery by the same surgeons. The patients had primary malignant tumors of the pelvis, and underwent limb-sparing resections between 1985 and 2000. The surgical method utilized threaded Steinman pins with bone cement to fill areas of bone loss that could not be reconstructed with autoclaved autograft. A constrained polyethylene acetabular component is cemented into this bed. Three outcome measures were evaluated: survival, function, and pain.
*Results:* Twelve patients had chondrosarcoma, two had alveolar soft parts sarcoma, and one had osteosarcoma. According to the Enneking-Dunham classification, there were 10 type II resections, and one each of types I, I/II, I/II/III, II/III, and III. There were 9 living and 6 deceased patients. For living patients, follow-up averaged 75 months (range 26 to 164) and for deceased patients, follow-up averaged 28 months (range 12 to 46). For chondrosarcoma patients, the local recurrence rate was 25% (3/12). All patients except one were ambulatory; 9 were able to walk without an assistive device. According to the MSTS Rating System, scores averaged 23/30 for living patients (range 10 to 27). Eleven patients were community ambulators and narcotics were used occasionally by only 2 patients. Surgical complications included 2 prosthetic dislocations, 2 wound hematomas, and 1 perioneal nerve palsy.
*Conclusion:* Reconstruction of massive pelvic defects with cement, rebar, autoclaved autograft, and constrained total hip replacements is a technique that compares favorably with other methods that have been reported. Functional outcome in terms of ambulatory capacity is substantially better than has been noted historically. The time required for independent gait is similar to recovery from routine total hip arthroplasty. Moreover, this method limits leg length discrepancy, while producing a favorable cosmetic result.

### 020 Curative Treatment and Effective Palliation with Hemipelvectomy—A Useful Operation: Modern Concepts Based on 20 Years of Experience

James C Wittig[1], Jacob Bickels[2], Felasfa Wodajo[1], Kristen L. Kellar-Graney[1], Yehuda Kollender[2], Isaac Meller[2], Martin M Malawer[1]
*[1]Division of Orthopedic Oncology Washington Cancer Institute Washington Hospital Center, [2]National Unit of Orthopedic Oncology Tel-Aviv Sourasky Medical Center*

*Objective:* The purpose of this study is to analyze the indications, describe the surgical technique, and report the oncological and functional outcomes and complications associated with hemipelvectomy.
*Methods:* Retrospective analysis of 44 patients (23 females, 21 males) treated with a hemipelvectomy between 1981 and 2001. Diagnoses: 14 pelvic and 9 proximal femur bone sarcomas, 3 metastatic carcinomas to the pelvis, 8 thigh soft tissue sarcomas, 1 soft tissue metastatic carcinoma, and 9 palliative (infection, trauma). Types of Hemipelvectomy: 13 classical, 21 modified, 6 extended and 4 anterior flap. Tumor Stages: IB (2), IIB (20), III (8), benign aggressive (1), stage IV lymphoma (2), metastatic carcinomas (5).
*Results:* Follow-up: 2 mos-181 mos (mean: 50.4 mos). There were 19 patients NED, 2 AWD, and 23 patients deceased. In the subset of patients with tumors (n=35), twelve patients (34%) were alive without evidence of disease. There was one local recurrence. All patients treated for infection, and trauma were successfully treated. Thirty-nine patients (89%) were pain free. All patients, except one patient, were ambulatory with crutches; two patients used a prosthesis. Four (10%) patients reported mild phantom limb pain. The most common complication was skin flap necrosis (8 patients); all were treated successfully with surgical debridement. Additional complications included: one perirectal abscess and two patients with urinary and/or erectile problems. There were no deaths due to intraoperative hemorrhage.
*Conclusion:* We recommend hemipelvectomy for curative treatment and palliation of selected pelvic and peripelvic tumors. It is a useful procedure when no limb sparing surgical option exists. It is also effective in certain instances of life threatening pelvic/lower extremity infection and trauma. Skin flap necrosis can be managed effectively. The incidence and severity of postoperative causalgia syndrome and urinary/bowel problems is minor. Survivors resume a functional life.

DOI: 10.1080/13577140120097058

**Young Investigator Award Winner**

### 016 Bugged Out? Infection and Endoprosthetic Replacements

Lee M Jeys, Raj Suneja, Vishnu Prasad, Robert Grimer, Simon R Carter, Roger Tillman
*Royal Orthopaedic Hospital Oncology Service, Royal Orthopaedic Hospital*

*Objective:* Endoprosthetic replacement (EPR) following surgical excision of bone tumours is now common practice. They allow rapid restoration of function, are readily available and have good long term functional scores. However, one of the major complications of EPRs is infection and this can have disastrous consequences. It requires prolonged and expensive treatment, and may result in decreased function, with even a possibility of amputation. This paper aims to investigate the cause of infection, management and its sequelae.
*Methods:* Prospective data is collected on all patients seen in our centre and stored on a database. Information collected includes demographic data, diagnosis, site, treatment (including adjuvant), complications, and outcomes. Records of over 10,000 patients have been kept over 34 years. Data was analysed on all patients who had undergone EPRs, in order to identify any infection, its management and outcome. Factors such as operating time, intra-operative blood loss, adjuvant therapy, type of prosthesis (extendable or standard) were investigated. Outcomes of treatment options were also evaluated.
*Results:* Over 1260 patients have undergone EPR in our center over 34 years. This gives a total follow up time of over 6500 patient years. Over 200 (16%) patients have been diagnosed with deep infection in their prosthesis (as defined by a positive culture in a clinically infected prosthesis) and of these 38 (25%) required amputations for uncontrollable infection. The commonest procedure for infection control was 2 stage revision. Common organisms involved in infection were *Staphylococcus aureus*, *Streptococcus viridians* and epidermidis.
*Conclusion:* Infections are one of the most serious complications to affect EPRs. Treatment can be difficult and prolonged. If early infection is diagnosed immediate lavage and IV antibiotics are recommended. Otherwise 2 stage revision is the only reliable method for limb salvage. Prevention must be the key to reducing the incidence of this serious complication.

### 056 Margin Width has Prognostic Significance for Extremity / Truncal Sarcoma

Mark D. McKee, Dong Feng Liu, Deborah Driscoll, John J. Brooks, John F. Gibbs, William G. Kraybill
*Roswell Park Cancer Institute*

*Objective:* Following resection of soft tissue sarcoma (STS), the presence of positive margins is associated with local recurrence and inconsistently reported to predict distant failure or shorter survival. Margin width is not emphasized in treatment planning or outcome analyses. We examined the prognosis of patients with wide margins (10+ mm), close margins (1-9 mm), and positive margins (0 mm).
*Methods:* Patients undergoing resection of primary extremity/ truncal sarcoma were evaluated for clinico-pathological features predicting outcome via retrospective review of charts and tumor specimens. Only patients with residual tumor available for pathologic review were included.
*Results:* Among 111 patients, tumors were predominantly intermediate to high grade (95), larger or equal to 5 cm in size (84), and deep (90). Treatment included wide excision or amputation for 110. High risk patients with positive or negative margins received adjuvant radiation (RT) (42 patients) and/or adjuvant chemotherapy (CT) (37 patients). Margin status: The margin width was 10+ mm in 53 patients (48%), 1-9 mm in 45 patients (40%), and 0mm in 13 patients (12%). Margins of 0 mm and 1-9 mm were more common among tumors of larger size (p=.008), and deep location (p=.01). More grade 1 tumors had 0 mm margins, and more grade 2 tumors had 10+ mm margins (p=.02). Age, gender, stage, site, necrosis, mitotic rate, resection type, and RT were similar among groups. Multivariate analysis: In a model including age, gender, grade, size, depth, stage, histology, tumor location, type of resection, RT, CT, and margin status, independent predictors of poor disease-free survival included higher grade (p=.009), larger size (p=.003), and decreased margin width (p=.007). Independent predictors of poor overall survival included higher grade (p=.001), larger size (p=.002), and deep location (p=.05).

### 106 Gene-Expression Profiles in Chondrosarcomas and Chordomas

Thomas F. DeLaney[1], Brian Seed[2], Ramnik Xavier[2], Andrew L. Rosenberg[3], G. Petur Nielsen[3], Francis J. Hornicek[4], Norbert J. Liebsch[1], John E. Munzenrider[1], Herman D. Suit[1]
[1]*Dept. of Radiation Oncology, Massachusetts General Hospital,* [2]*Dept. of Molecular Biology, Massachusetts General Hospital,* [3]*Dept. of Pathology, Massachusetts General Hospital,* [4]*Dept. of Orthopedic Surgery, Massachusetts General Hospital*

*Objective:* Outcome analysis of more than 200 patients each with low grade chondrosarcoma and chordoma of the skull base following high dose proton beam radiation treatment has revealed a very large difference between these two pathological tumor types. Namely, the 10 year local control results were 95% and 45% respectively. These tumors were of approximately the same size, anatomic site and received the same radiation doses. Further, they were managed by the same clinicians and physicists. We hypothesize that there are important differences in the radiation sensitivity of the constituent tumor cells of these tumors. Our research plan is to assess differences in expression profiles of those genes involved in repair of DNA and angiogenesis. This information is expected to help explain the differences in radiation sensitivity of these tumors.
*Methods:* RNA from samples of 10 chordomas and 10 chondrosarcomas will be compared with a microarray of complementary DNA clones which included panels of genes known to be involved in DNA repair or angiogenesis. Statistical analyses will be used to identify a set of genes that could distinguish the very different clinical response of chondrosarcomas and chordomas to radiotherapy.
*Results:* Tumor samples from 10 patients with chordomas and 10 patients with chondrosarcomas have been obtained. Initial RNA extraction has been successful and cDNA arrays of genes associated with angiogenesis, cellular stress, and cancer metastasis have been designed. Gene expression profiles for the tumors are in progress. Results of the analysis will be presented.
*Conclusion:* Gene expression profiles for chondrosarcomas and chordomas may elucidate differences in clinical behavior.

**Young Investigator Award Winner**

### 131 Inhibition of PDGF Receptor Tyrosine Kinases By STI-571 In Human Soft-Tissue Sarcoma

Jonathan C. Trent, Jonathan Hannay, Lan Li, Shreyaskumar Patel, Robert S. Benjamin, Raphael E Pollock, Di-Hua Yu
*The University of Texas, M. D. Anderson Cancer Center*

*Objective:* Abnormalities in signal transduction pathways are common in soft tissue sarcomas. Because these are rare tumors that also respond poorly to standard chemotherapy and bear a 50% 5-year mortality rate, we investigated the possible therapeutic benefits of the tyrosine kinase inhibitor STI-571 in soft-tissue sarcomas. The objectives of our laboratory investigation are (1) to characterize the anti-tumor activity of STI-571 alone and with doxorubicin, *in vitro*; (2) to determine whether the inhibition of signal transduction molecules by STI-571 causes cell cycle arrest, induction of apoptosis, inhibition of the invasive phenotype and/or abrogation of angiogenesis; (3) to determine the anti-tumor activity of STI-571 alone and in combination with doxorubicin in human tumor xenografts.

*Methods:* We initially screened 15 soft-tissue sarcoma cell lines for expression of PDGF-R. Western blot analysis identified 12 that expressed high levels and 3 that expressed low levels. We have investigated the efficacy of STI-571 (0.4-20micromolar) alone and combined with doxorubicin(0.1-10micromolar) in the rhabdomyosarcoma cell line A204. Kit, PDGF-R, Map kinase, and Akt-1 signaling were evaluated by immunoblot. Proliferation assays were performed with a 48 hour exposure to incremental drug concentrations and viability assessed by MTS reagent. TUNEL assay and cell cycle analysis were performed by flow cytometry. Soft agar colony formation assay was performed to assay for anchorage independent growth. Endothelial cell proliferation rate in response to conditioned media from STI-571 treated A204 cells will be assessed. Zymogen invasion assay will be performed as an *in vitro* assessment of the invasive phenotype. SCID mice will be injected with A204 cells prior to treatment with doxorubicin alone, STI-571 alone, or the combination.

*Results:* Sarcoma cells treated with doxorubicin, STI-571, or the combination, exhibit a 40%, 20%, and 60% absolute reduction in percent viable cells, respectively. STI-571 increased the relative percentage of cells in G1 (by 57%) and decreased S-phase (by 63%) when compared to untreated cells. Apoptosis increased from 4% to 15% after treatment. Doxorubicin treatment did not induce cell cycle arrest, but induced apoptosis from a baseline of 5% to 50%. STI-571 treatment resulted in a decrease (48%) in the number of colonies established by soft agar growth. Endothelial cell proliferation assay, Zymogen invasion assay, and *in vivo* therapy with STI-571 experiments are ongoing.

*Conclusion:* *In vitro* results suggest an additive, non-overlapping effect of doxorubicin+STI-571, indicating a potentially useful combination. Together, these efforts should result in new mechanism-driven therapies that are efficacious in soft-tissue sarcoma.

**130 Expression Profiling of Osteosarcoma Biopsies and Metastases with cDNA Microarrays**

Luca Sangiorgi[1], Giuliana Alessandra Gobbi[1], Enrico Lucarelli[1], Francesca Scrimieri[1], Piero Picci[1], Paul Meltzer[1]
*[1]Lab. Oncology Research, Rizzoli Orthopedic Institute, [2]Section of Molecular Genetics, Cancer Genetics Branch, National Human Genome Research Institute, National Institutes of Health*

*Objective:* Expression profiling with cDNA microarrays offers the possibility to determine global patterns of gene expression tumor specimens which can be used for diagnostic classification, the identification of subsets as well as the elucidation of oncogenic pathways and novel therapeutic targets.

*Methods:* We have explored the potential of applying this technology to osteogenic sarcoma. 14 primary osteosarcomas and 8 osteosarcoma pulmonary metastases were analyzed by hybridiation to cDNA microarrays. For comparison, five Ewing's sarcoma specimens, and normal lung tissue were also analyzed.

*Results:* We determined that high quality results could be obtained from osteosarcoma specimens despite the presence of extracellular matrix material in these specimens. A distinct pattern of gene expression for osteosarcoma was readily identified. This osteosarcoma specific gene expression signature was detectable in pulmonary metastases. Additionally, lists of genes which discriminate osteosarcoma and Ewing's sarcoma were developed. Inspection of these gene lists identify a number of signal transduction and cell cycle genes utilized differentially by these two cancers.

*Conclusion:* Several genes expressed by osteosarcoma have potential for development as targets for immunohistochemical detection for diagnostic purposes.

